# A computer vision method to evaluate tumor-infiltrating lymphocytes and multiparametric modeling of neoadjuvant systemic therapy response in breast cancer

**DOI:** 10.1177/17588359261417762

**Published:** 2026-02-20

**Authors:** Mateusz Bielecki, Fang-I Lu, Angeline Vo, Eileen Rakovitch, Katarzyna J. Jerzak, Roberto Salgado, Raffi Karshafian, William T. Tran

**Affiliations:** Biological Sciences Platform, Sunnybrook Research Institute, 2075 Bayview Ave., Room TB 097, Toronto, ON M4N 3M5, Canada; Department of Physics, Toronto Metropolitan University, Toronto, ON, Canada; Department of Laboratory Medicine and Molecular Diagnostics, Sunnybrook Health Sciences Centre, Toronto, ON, Canada; Biological Sciences Platform, Sunnybrook Research Institute, Toronto, ON, Canada; Biological Sciences Platform, Sunnybrook Research Institute, Toronto, ON, Canada; Department of Radiation Oncology, University of Toronto, Toronto, ON, Canada; Division of Medical Oncology and Hematology, Odette Cancer Centre, Sunnybrook Health Sciences Centre, Toronto, ON, Canada; Department of Pathology, ZAS Hospitals, Antwerp, Belgium; Department of Physics, Toronto Metropolitan University, Toronto, ON, Canada; Institute for Biomedical Engineering, Science and Technology, a partnership between Toronto Metropolitan University and St. Michael’s Hospital, Toronto, ON, Canada; Biological Sciences Platform, Sunnybrook Research Institute, Toronto, ON, Canada; Department of Radiation Oncology, University of Toronto, Toronto, ON, Canada

**Keywords:** advanced breast cancer, machine learning, neoadjuvant systemic therapy, prediction models, tumor-infiltrating lymphocytes

## Abstract

**Background:**

Neoadjuvant systemic therapy (NST) is often used to treat locally advanced breast cancer (BC) or patients with early-stage BC at high risk for micrometastatic spread. Pathological complete response (pCR) to NST in BC is associated with excellent prognostic outcomes; however, rates vary significantly. Tumor-infiltrating lymphocytes (TILs) are associated with NST response, suggesting potential as predictive biomarkers.

**Objective:**

To develop a computer vision approach to quantify spatial TIL parameters and a multiparametric machine learning (ML) model for predicting NST response.

**Design:**

Retrospective, single institution study of 411 BC patients, combining clinical and graph-level pre-treatment histopathology data to predict response to NST using ML.

**Methods:**

Pre-treatment core needle biopsies were prepared, stained with hematoxylin and eosin, and digitized into whole slide images. Convolutional neural networks were applied to segment and classify regions of invasive carcinoma and TILs. Spatial features were extracted based on the coordinates of the TILs within invasive regions, including metrics from Delaunay triangulation, Voronoi diagram analysis, and minimum spanning trees, as well as features capturing cell density and nuclear count. Clinicopathological features were incorporated to support multiparametric modeling. Multiple ML classification models were trained to predict pCR. Logistic regression, K-nearest neighbor, support vector, random forest, Gaussian Naïve Bayes, and extreme gradient boosting models were tested, and model performances were reported.

**Results:**

ML models using clinical and graph-based features achieved high predictive accuracy. The best performing graph feature model reached an area under the receiver operating characteristic curve (AUC) of 0.924. Ensemble models integrating clinical and graph features showed the highest performance, with an AUC of 0.955. Notably, for triple-negative BC, significant differences in predictive performance were demonstrated between clinical and graph feature models (*p* = 0.026) and between clinical and ensemble models (*p* = 0.006).

**Conclusion:**

Multiparametric modeling utilizing clinicopathological and graph features obtained from TILs is associated with pCR in BC patients treated with NST.

## Introduction

Breast cancer (BC) is the most prevalent malignancy in women and the second leading cause of cancer-related death.^1,2^ Patients with locally advanced breast cancer (LABC) and those with stage II–III triple-negative (TNBC) or human epidermal growth factor receptor 2-positive (HER2+) BC are generally treated with neoadjuvant systemic therapy (NST). A pathological complete response (pCR), that is, no residual invasive carcinoma in the breast and axillary lymph nodes (ypY0/is ypN0), is the desired treatment outcome since it is associated with excellent disease-free survival (DFS) and overall survival (OS).^3,4^ A pCR is achieved in up to 65% of TNBC patients receiving multi-agent chemotherapy plus immunotherapy, and similarly high pCR rates are observed in HER2+ disease treated with HER2-targeted therapy and chemotherapy. However, the pCR rate is substantially lower and more variable among other subtypes.^3,5,6^ Predicting pCR a priori could enable personalized and tailored treatments.

Several studies have shown that NST response is mediated by immunoregulatory factors, such as tumor-infiltrating lymphocytes (TILs).^7,8^ Experimental works describe a signaling cascade promoted by tumor cells releasing tumor antigens. Subsequently, an activation of TILs can drive immunogenic cell death.^9^ Pre-therapeutic TILs predict NST response, particularly in TNBC^10–13^ as assessed by quantifying stromal TILs (sTILs), although intratumoral TILs (iTILs) have also been studied previously.^14,15^ TILs are commonly assessed manually using a visual TILS assessment (VTA). An early study by Yamaguchi et al.^16^ evaluated 68 BC patients for responses to NST using a TILs assessment method proposed by Aaltomaa et al.^17^ Lymphocytic infiltrates in the tumor margin and stroma were visually assessed and graded (i.e., weak/absent, moderate, or dense), then a binary classification of low/high TILs was used for statistical analysis. Univariate analysis showed a significant association between high TIL infiltration (*p* < 0.0001), high-grade (*p* = 0.0206), and hormone receptor (HR)-/HER2+ status (*p* = 0.014) with pCR. Multivariate principal component regression analysis showed that high TILs were a significant independent predictor of pCR (odds ratio = 4.7; *p* < 0.0001).^16^ A study by García-Martínez et al. investigated 121 early-BC patients treated with NST. Pre-treatment TIL populations were grouped according to immune cell profiles using immunohistochemistry. A hierarchical clustering analysis was conducted on six immune markers (CD4, CD8, CD3, CD20, FOXP3, and CD68). The results showed that immune clusters with low CD8, high CD4, high CD20, and high CD68 infiltration were associated with high-grade tumors and were prevalent in 58% of patients who achieved a pCR.^18^ In addition, a comparison of mean TIL cell count per area (mm^2^) showed a significant difference in CD3, CD4, and CD20 infiltrates between pCR and non-pCR patients.^18^ Denkert et al. conducted a pooled analysis of 3771 patients from 6 randomized BC trials. The landmark study assessed sTILs from pre-therapeutic core needle biopsies (CNBs) using standardized methods outlined by the International Immuno-Oncology Biomarker Working Group (IBWG). TILs were evaluated according to the percentage of tumor stromal area occupied. TIL levels were analyzed as a continuous variable (in 10% increments) and as an ordinal variable, categorized as low (0%–10%), intermediate (11%–59%), or high (⩾60%). The results showed that higher TIL concentration predicted NST response; tumors with high concentration of TILs (i.e., ⩾60%) had the highest rate of pCR (*p* < 0.001). Multivariate analysis using TILs as a continuous parameter showed an association between increasing TIL concentrations and the rate of pCR.^15^ Notably, high TILs and pCR were more prevalent in TNBC (50% of patients) and HER2+ BC (48% of patients) versus the luminal-HER2-negative subtype (28% of patients).^15^

More recent studies also corroborate the association between TILs and NST response. Liang et al. investigated the spatial importance between TILs and tumor cells in BC patients treated with NST. Immune cells were evaluated according to their proximity to tumor cells using Euclidean distance. CD8+ T-cells within 20 µm of tumor cells demonstrated significant correlation to pCR, DFS, and OS outcomes. Higher percentages of CD8+ T-cells to tumor cells were evaluated in intratumoral (*p* *<* 0.001) and stromal (*p* = 0.003) regions for patients with pCR versus non-pCR. This was more pronounced in TNBC.^19^ Russo et al. evaluated pathological response in 187 LABC patients treated with NST. A pCR was observed in 58.5% of TNBC and HER2+ patients with high pretreatment TIL infiltrates (>30%).^20^ Khoury et al.^21^ proposed a model using pre-treatment BC core biopsies; sTILs were assessed using the IBWG 2014 guidelines. The iTILs parameter was calculated semi-quantitatively; a 4-point infiltrate grade (0–3) was assigned and multiplied by the percentage of tumor contained within each score. This yielded a so-called *H*-Score, which ranged from 0 to 300. There was a significant correlation between sTILs and iTILs (Spearman *r* = 0.707; *p* < 0.001). In multivariate analysis, sTILs and iTILs were independent predictors of pCR in TNBC but not HER2+ BC.^21^ Taken together, previous VTA studies have utilized various TIL assessment methods, modeling approaches, and significance in sTILs and iTILs to predict NST response. Limitations include errors in reproducibility, inter-observer variability, and the lack of incorporation of spatial data within this dynamic immune environment.^14,22,23^ Current research into digital pathology methodologies is poised to address these challenges.

Digital pathology and recent advances in artificial intelligence (AI) have allowed for increased systemization of histological analysis, permitting the extraction and handling of high-dimensional data that would not be possible with manual analysis.^14,24^ Quantitative analysis of TILs on whole slide images (WSIs) allows one to identify unique features and complex interactions between cancer and immune cell types.^25^ Machine learning (ML) algorithms have been implemented in predictive and prognostic applications within BC.^25–27^ ML models can utilize large datasets and identify the most relevant features to maximize outcome prediction. Herein, there is an opportunity to identify and extract spatial information from TILs in BC patients to predict pCR. Here, we evaluate the spatial and density-related distribution of TILs and use a multiparametric model to predict pCR in BC patients treated with NST.

## Methods

### Patient population and ground truth labels

**Figure 1.**
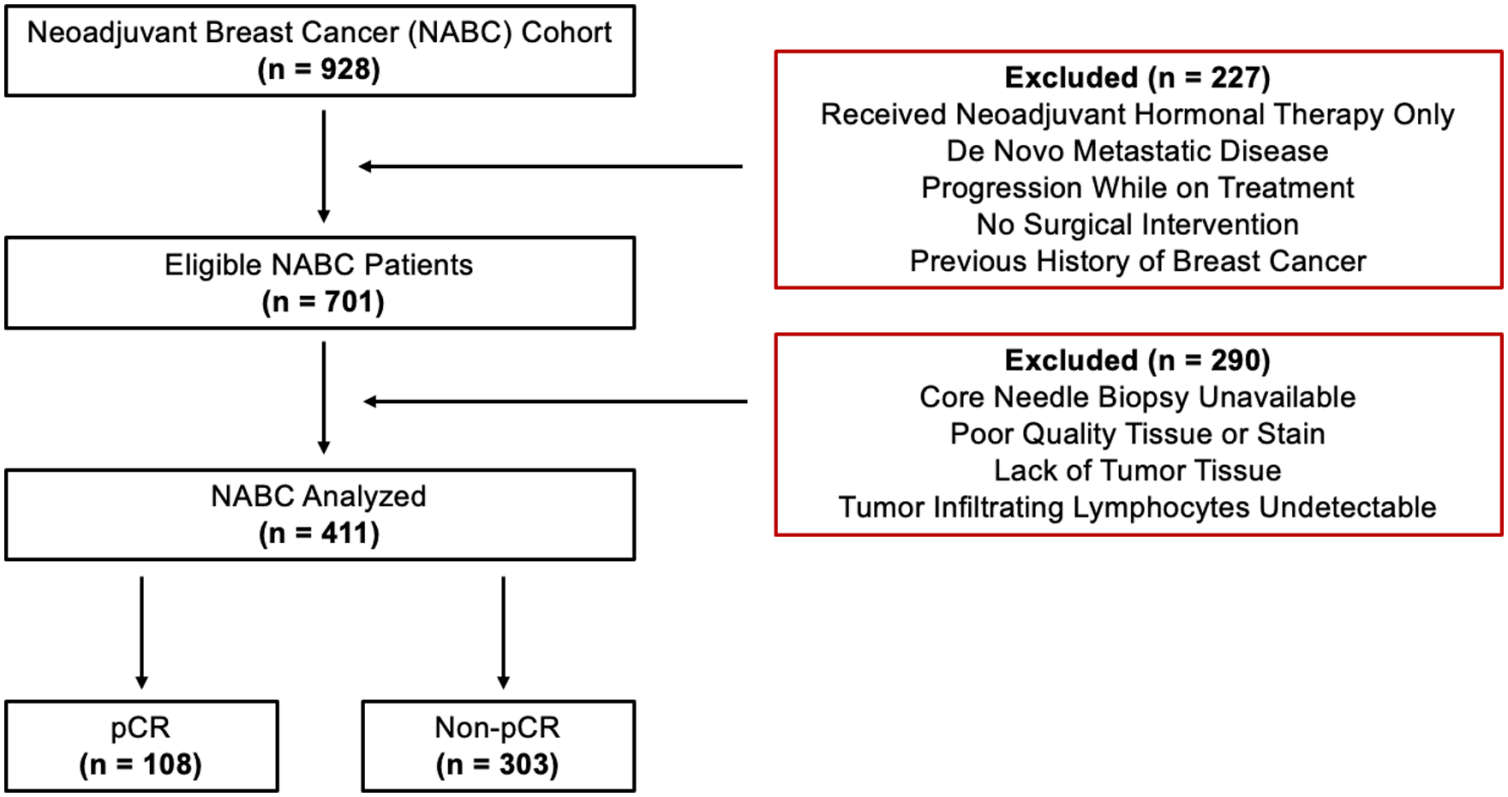
Flow diagram of the patient cohort, with bolded numbers indicating the number of indviduals at each stage. A total of 411 patients were included in the machine learning analysis.

This retrospective study included patients treated at a single Canadian cancer treatment center between 2009 and 2021. The reporting of this study conforms to the TRIPOD statement for reporting of multivariable prediction models for individual prognosis and diagnosis, and the completed checklist is provided (Supplemental Material).^28^ The Institutional Ethics Board approved this research. Subjects were identified using an electronic medical record (EMR) system. Patients who were pathologically confirmed with invasive BC treated with NST were included. The inclusion criteria comprised patients treated with an anthracycline–taxane backbone chemotherapy. Patients with HER2+ BC were given trastuzumab during the taxane block sequence. Docetaxel, carboplatin, trastuzumab, and pertuzumab treatment was not included in this analysis since Pertuzumab was not approved for routine treatment within the study analysis window. Patients with TNBC who were treated with pembrolizumab according to the KEYNOTE-522 regimen were also excluded on the basis of insufficient numbers within the analysis period of this study. Clinical data were extracted using the EMR system (Table 1). Patients were selected for analysis based on inclusion/exclusion criteria (Figure 1).

**Table 1.** Patient clinicopathological characteristics.

Patient clinicopathological characteristics	Study cohort (n = 411)		
	pCR (n = 108) n (%)	Non-pCR (*n* = 303) *n* (%)	*p*-Value
Age			
Mean Age ± SD (years)	50.8 ± 10.4	51.2 ± 11.4	0.748
⩽50 years	56 (51.9)	143 (47.2)	0.406
>50 years	52 (48.1)	160 (52.8)	
Menopausal status			
Pre	48 (44.4)	132 (43.6)	0.610
Post	53 (49.1)	141 (46.5)	
Peri	7 (6.5)	29 (9.6)	
Laterality			
Left	55 (50.9)	141 (46.5)	0.433
Right	53 (49.1)	162 (53.5)	
Neoadjuvant chemotherapy			
FEC-D	35 (32.4)	134 (44.2)	0.028*
AC-T	72 (66.7)	169 (55.8)	
Other	1 (0.9)	0 (0.0)	
Anti-HER2 therapy			
Yes	69 (63.9)	91 (30.0)	~0.000*
No	39 (36.1)	212 (70.0)	
Receptor status			
ER+	38 (35.2)	226 (74.6)	~0.000*
ER−	70 (64.8)	77 (25.4)	
PR+	23 (21.3)	195 (64.4)	~0.000*
PR−	85 (78.7)	108 (35.6)	
HER2+	70 (64.8)	92 (30.4)	~0.000*
HER2−	38 (35.2)	211 (69.6)	
Tumor size			
Mean size ± SD (mm)	39.0 ± 25.5	47.6 ± 26.9	~0.000*
Clinical T stage			
1	15 (13.9)	26 (8.6)	0.007*
2	73 (67.6)	169 (55.8)	
3	20 (18.5)	100 (33.0)	
4	0 (0.0)	8 (2.6)	
Clinical N stage			
0	47 (43.5)	79 (26.1)	0.005*
1	51 (47.2)	183 (60.4)	
2	7 (6.5)	25 (8.3)	
3	3 (2.8)	16 (5.3)	
Node status			
Positive	61 (56.5)	224 (73.9)	0.001*
Negative	47 (43.5)	79 (26.1)	
Inflammatory breast cancer			
Yes	6 (5.6)	25 (8.3)	0.362
No	102 (94.4)	278 (91.7)	
Nottingham grade			
1	2 (1.9)	17 (5.6)	~0.000*
2	22 (20.4)	159 (52.5)	
3	80 (74.1)	127 (41.9)	
Histology			
IDC	105 (97.2)	276 (91.1)	0.013*
ILC	0 (0.0)	22 (7.3)	
IMC	3 (2.8)	5 (1.7)	
BRCA gene mutation			
Positive	7 (6.5)	17 (5.6)	0.724
Negative	100 (92.6)	286 (94.4)	

* Statistically significant difference (*p* < 0.05).

AC-T, adriamycin cyclophosphamide and Taxol; ER, estrogen receptor; FEC-D, 5-fluorouracil epirubicin cyclophosphamide and docetaxel; HER2, human epidermal growth factor receptor 2; IDC, invasive ductal carcinoma; ILC, invasive lobular carcinoma; IMC, invasive mammary carcinoma; pCR, pathological complete response; PR, progesterone receptor.

As part of the patient’s standard of care, ultrasound-guided CNB were retrieved during the patient’s diagnostic workup. Samples were collected by a board-certified radiologist using a 14-gauge needle, fixed in formalin, and then embedded in paraffin. The formalin-fixed paraffin-embedded blocks were cut into 4 µm sections and prepared onto glass slides. The samples were stained with standard hematoxylin and eosin (H&E) techniques, and biomarker (ER/PR/HER2) status was evaluated using standard processing guidelines by the College of American Pathologists. Board-certified pathologists reviewed the pre-treatment core biopsies to confirm a BC diagnosis. The post-treatment excisions were used to quantify the presence of invasive and in situ residual disease following NST. Pathological response was assessed using the Residual Cancer Burden Index (RCBI) by pathologists and reported in EMR as either pCR or residual disease.^29^ For this study, response data were extracted as binary ground truth labels: (1) pCR (i.e., RCBI = 0) or (2) non-pCR (non-pCR, i.e., residual disease, RCBI = 1–3).

In total, 928 BC patients were initially identified within the study time interval (2009–2021). After screening, 411 subjects satisfied the study criteria with pre-treatment CNBs and were eligible for analysis (Figure 1). To reduce image irregularities (blurriness or distortion), quality control measures, such as a manual review of the specimen for fragmentation, degradation, and orientation, were carried out before scanning. The slides were digitized (scanned) using a pathology imaging scanner (TissueScope LE; Huron Digital Pathology Inc., St. Jacobs, ON, Canada). All of the slide images (WSIs) were captured at 40× magnification.

### Computer vision to identify TILs

A computer vision pipeline was developed to analyze the tumor images. WSIs underwent preprocessing to localize the tumoral regions of interest (ROIs) containing lymphocytes in the pre-treatment core biopsy specimen. An Otsu thresholding binary classification technique was implemented to train the model to separate the foreground tissue from the slide background. A contoured boundary delineated the core biopsy section and was used to create smaller image tiles (750 × 750 pixels) with no overlap and a maximum background of 10%. Tiles were stain-normalized to remove color variations. The images were subsequently input into a modified VGG-19 convolutional neural network (CNN), which output probability maps that predicted the areas of the invasive tumor bed.^27,30^

**Figure 2.**
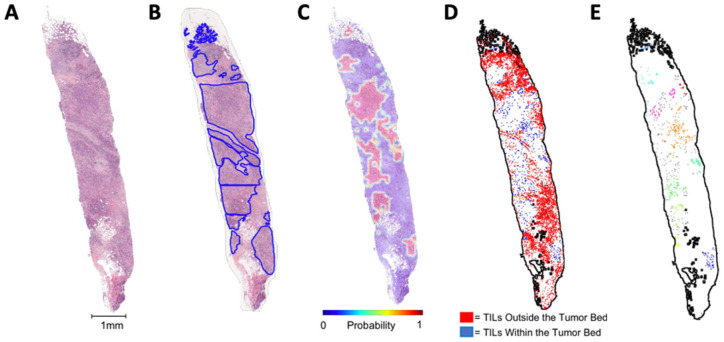
Representative WSI pre-processing and segmentation. (a) H&E image. (b) Ground-truth annotation. (c) CNN generated heatmap of tumor bed. (d) TIL density map. (e) Clustered TILs within the tumor bed. CNN, convolutional neural network; H&E, hematoxylin and eosin; TIL, tumor-infiltrating lymphocytes; WSI, whole slide images.

Segmentation and classification of nuclei, including epithelial, lymphocytic, macrophagic, and neutrophilic, were completed using HoVer-Net architecture.^31^ This algorithm was pre-trained on the Multi-Organ Nuclear Segmentation and Classification (MoNuSAC) dataset to augment the performance of the internal dataset.^32^ The MoNuSAC dataset includes cell-level annotations created by expert pathologists on digitized WSIs of breast, prostate, kidney, and lung tissue at 40× magnification.^31^ HoVer-Net was tasked to classify centroids and boundaries of the internal dataset, where only lymphocyte information was retained for analysis (Figure 2). The deep learning (DL) architecture is composed of three different branches that (1) distinguish whether pixels belong to background or nuclei, (2) resolve cell separation through the determination of horizontal and vertical boundaries, and (3) classify each cell. Lymphocyte annotations were subsequently verified by a board-certified pathologist. To identify lymphocytes located within the tumor bed, the centroids of each cell were compared to the *X* and *Y* coordinates of tumor bed tiles.

### Multiparametric feature extraction

#### Patient and clinical features

Patient and clinical information were captured. These included the patient’s age, menopausal status, breast laterality, systemic therapy regimen, biomarker status, radiological tumor size, TNM (tumor, node, metastasis) information, tumor grade, histological type, and germline BRCA gene mutation status. A summary of the parameters is presented in Table 1.

#### Spatial (graph) feature extraction of TILs

**Figure 3.**
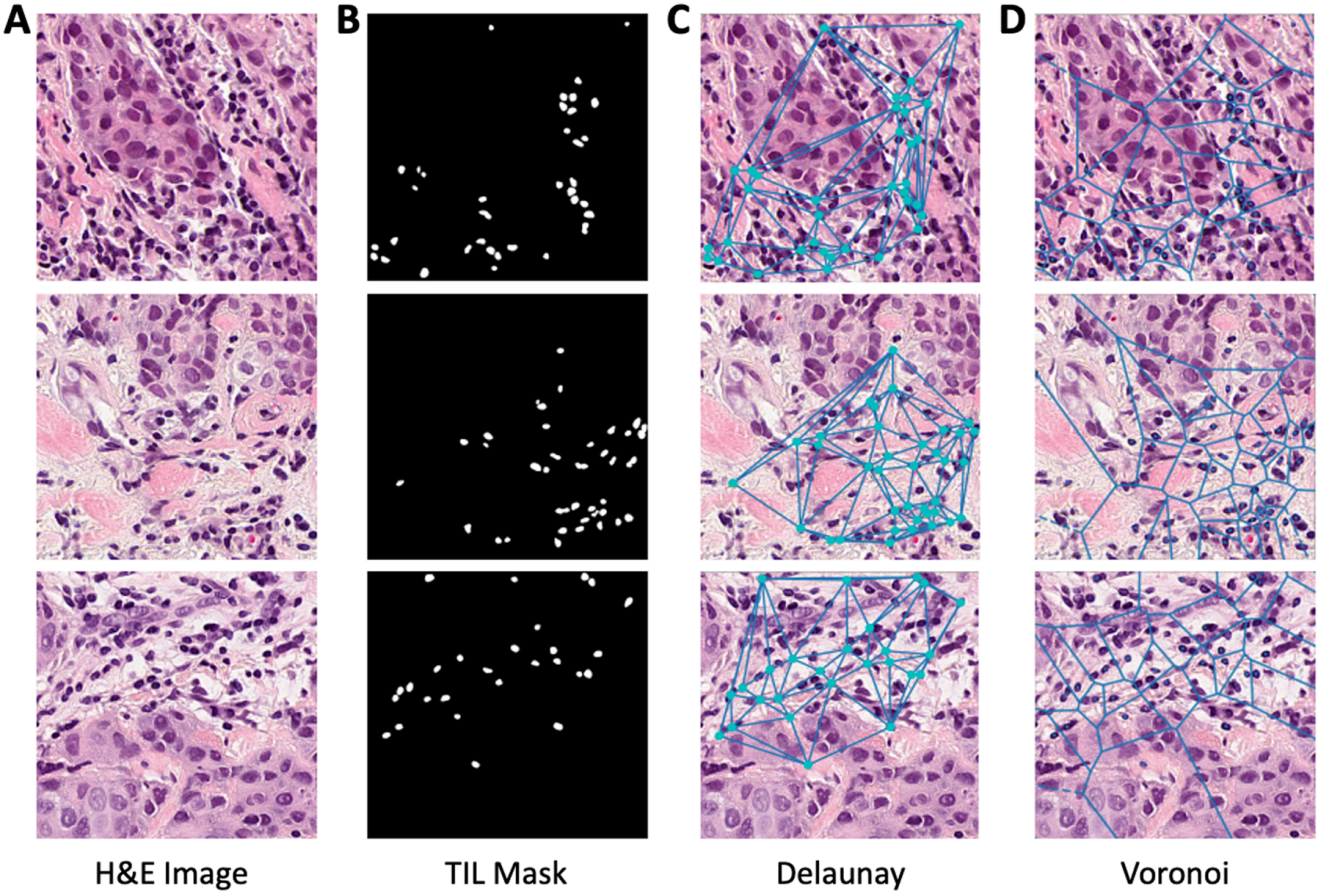
Representative image of lymphocytes within H&E tile images. (a) H&E tile image. (b) Generated mask of TILs within tiles. (c) Delaunay triangulation of TILs. (d) Voronoi diagram of TILs. H&E, hematoxylin and eosin; TIL, tumor-infiltrating lymphocytes.

As part of the multiparametric models, graph-level features were extracted based on the coordinates of the TILs within the invasive tumor bed. There were 371 spatial features calculated. The features included hierarchical density-based spatial clustering of applications with noise (*f* = 130), Voronoi features (*f* = 12), Delaunay features (*f* = 8), minimum spanning tree features (*f* = 4), density features (*f* = 216), and the TILs frequency (*f* = 1; Figure 3).^33^

### ML classification to predict NST response

Several ML models were developed utilizing a multiparametric approach. These included models based on clinical data, aggregated graph data only, and combined clinical and graph data (Ensemble). The ML algorithms used included the following: (i) Gaussian Naïve Bayes (GNB), (ii) K-nearest neighbor (KNN), (iii) logistic regression (LR), (iv) random forest classifier (RFC), (v) support vector machine (SVM), and (vi) extreme gradient boosting (XGBoost). These models were selected based on their versatility in handling various data distributions and types, simplicity in hyperparameter selection, and computational efficiency in model training and testing.^34,35^

Clinical and graph features were tested for multicollinearity to eliminate redundant features. Pearson and point-biserial correlation coefficients were used for continuous-continuous and continuous-dichotomous data, respectively, within the dataset. Pearson and Theil’s *U* tests were used for continuous–continuous and nominal–nominal variables for the clinical dataset. Identified variables that were less correlated with the dependent variable were removed. The training dataset was normalized using a *Z*-Score normalization, where mean and standard deviation values were retained to standardize the test subset.

We carried out two modeling experiments. In the first experiment (Experiment 1), the dataset underwent a train-test split to fit and evaluate ML models. This was completed by randomly splitting the entire cohort into 80% for training and 20% for model testing. For subgroup analysis, we conducted a secondary modeling exercise (Experiment 2A, 2B). Since the previous literature has shown that HER2+ and TNBC subtypes demonstrate various TIL activities, we generated a model using patients within these biomarker subgroups.^15,36,37^ A train-test ratio (80:20) was used in Experiment 2, that is, HER2+ BC (training: *n* = 44, test: *n* = 12) and TNBC (training: *n* = 68, test: *n* = 17). For ML, feature sets were aggregated in seven ways: per core method, maximum, minimum, mean, weighted-mean, median, and mean–minimum–maximum. A wrapper sequential forward feature selection (SFFS) technique was implemented to identify optimal feature combinations for each model. This was done using a stratified 10-fold cross-validation (CV) technique and repeated for 100 iterations within each model. Through the 100 iterations, features that showed the highest frequency were determined to be most important and were retained. To limit the effects of dimensionality, the number of features in each model was limited to a maximum of 1 feature for every 10 training samples.^38^ As such, HER2+ and TNBC models were limited to four and seven features, respectively. A stratified 10-fold CV technique with an integrated synthetic minor oversampling technique was used to train and tune the hyperparameters of each model with the optimal feature set and account for class imbalance. This was done using two hypertuning algorithms, Hyperopt or randomized grid search (RGS). The hyperparameters that optimized the area under the receiver operator characteristic (ROC) curve (AUC) score were retained.

Ensemble models were created by evaluating all possible clinical and graph-level feature combinations. One hundred different combinations were indicated using weighted aggregation between zero and one. These values weighted the aggregation function (mean) of the combined predicted probabilities for clinical and graph models. A value of zero indicated that the ensemble model weighted the clinical model at 100%, while a value of 1 indicated that the ensemble model weighted the graph model at 100%.

### Model evaluation

The performance of ML models was evaluated on the independent test set using the following metrics: prevalence, accuracy, sensitivity, specificity, F1-score, and AUC. Models were assessed using the features identified within SFFS and through hyperparameter tuning. The highest-performing models for each molecular subtype and associated features were retained. Pairwise comparisons of ROC curves were conducted using DeLong’s test to determine whether observed differences were statistically significant within each group.^39^ *p*-Values < 0.05 were statistically significant.

## Results

### Clinicopathological characteristics

A total of 411 patients were included in the analysis. HR-positive patients accounted for 270 (65.6%), HER2+ accounted for 56 (27.7%), and TNBC accounted for 85 (20.7%). In the full cohort (Experiment 1), *n* = 108 (26.2%) achieved a pCR, and *n* = 303 (73.8%) did not achieve a pCR (non-pCR group). In a subgroup analysis (Experiment 2), pCR was achieved by *n* = 37 (66.1%) HER2+ patients, and *n* = 30 (35.3%) TNBC patients.

The average age of subjects in this study was 51 years. The mean size of tumors at the time of diagnosis in the pCR and non-pCR groups was 39.0 mm + 25.5 mm and 47.6 mm + 26.9 mm, respectively. The NST regimens included a block-sequence regimen of either adriamycin cyclophosphamide and Taxol (dose-dense) or 5-fluorouracil epirubicin cyclophosphamide and docetaxel. All clinicopathological characteristics of the study subjects are presented in Table 1.

### ML prediction performances

**Figure 4.**
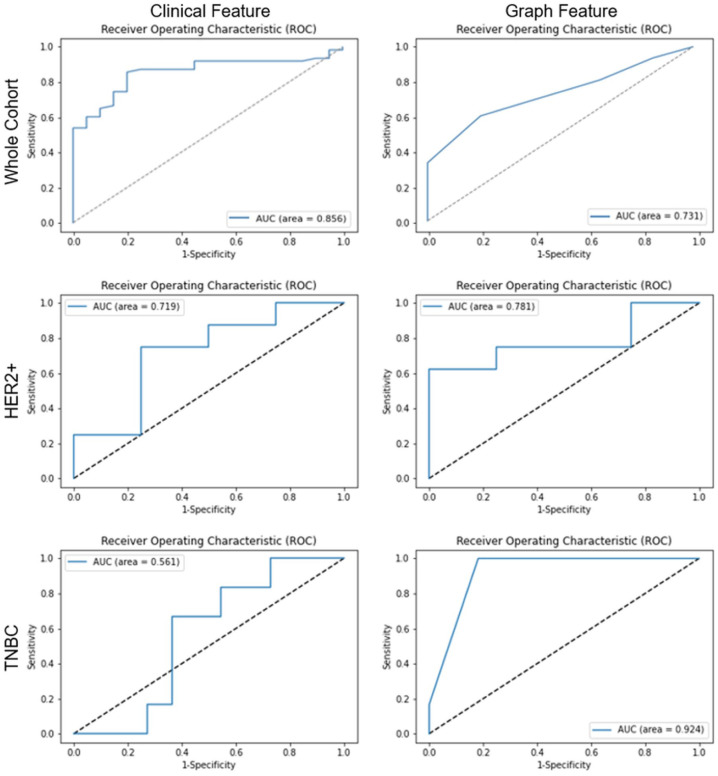
Represents the ROC AUC for the highest-performing clinical feature (left) and graph feature (right) models with optimized features for each molecular subtype. AUC ROC, area under the receiver operator characteristic curve.

ML prediction models were implemented to predict pCR versus non-pCR. Independent ML models were trained using clinical and graph features. Six different ML models were used in the final analysis. Model hyperparameters were tuned using RGS or Hyperopt. The highest-performing models were identified for each molecular subtype across clinical, graph, and ensemble-based ML models. Highest AUC performance scores varied with molecular subtype, ranging between 0.561 and 0.856 for the clinical models, 0.731 and 0.924 for the graph models, and 0.812 and 0.955 in ensemble models. ML model AUC and metrics are indicated in Table 2. Clinical and graph AUC curves are shown in Figure 4.

**Table 2.** Performance metrics for top-performing models.

Group	Model	Classifier	AUC	Acc	Pre	F1	Sn (%)	Sp (%)
Whole cohort	Clinical	XGBoost	0.856	0.843	0.917	0.894	87.3	75.0
	Graph	KNN_min_	0.731	0.650	0.904	0.724	60.3	80.0
	Ensemble	XGBoost + GNB_min_	0.881	0.759	0.977	0.815	69.8	95.0
HER2+	Clinical	LR	0.719	0.750	0.778	0.824	87.5	50.0
	Graph	KNN_median_	0.781	0.750	0.857	0.800	75.0	75.0
	Ensemble	RFC + KNN_median_	0.812	0.750	0.857	0.800	75.0	75.0
TNBC	Clinical	SVM	0.561	0.529	0.429	0.600	100	27.2
	Graph	KNN_mean_	0.924	0.882	0.750	0.857	100	81.8
	Ensemble	SVM + KNN_mean_	0.955	0.647	0.500	0.666	100	45.4

Ensemble models combine clinical and graph classifiers, respectively.

Acc, accuracy; AUC, area under the receiver operating characteristic curve; GNB, Gaussian Naïve Bayes; HER2, human epidermal growth factor receptor 2; KNN, K-nearest neighbor; LR, logistic regression; Pre, precision; RFC, random forest classifier; Sn, sensitivity; Sp, specificity; SVM, support vector machine; TNBC, triple-negative breast cancer; XGBoost, extreme gradient boosting.

The highest-performing ensemble model for each molecular subtype was evaluated. The whole cohort demonstrated an AUC of 0.881 (95% confidence interval (CI): 0.807, 0.950) with an accuracy of 0.759, whereas the HER2+ subtype yielded an AUC of 0.812 (95% CI: 0.501, 1.000) and an accuracy of 0.750. The TNBC ensemble model showed the highest performance overall with an AUC of 0.955 (95% CI: 0.827, 1.000), indicating an accuracy of 0.647. The whole cohort ensemble model that performed optimally on the test set combined the XGBoost clinical model with the GNB_min_ graph model. This ensemble model indicated a threshold value of 0.22. The HER2+ group combined the RFC clinical model with the KNN_median_ graph model, utilizing a threshold of 0.63.

**Figure 5.**
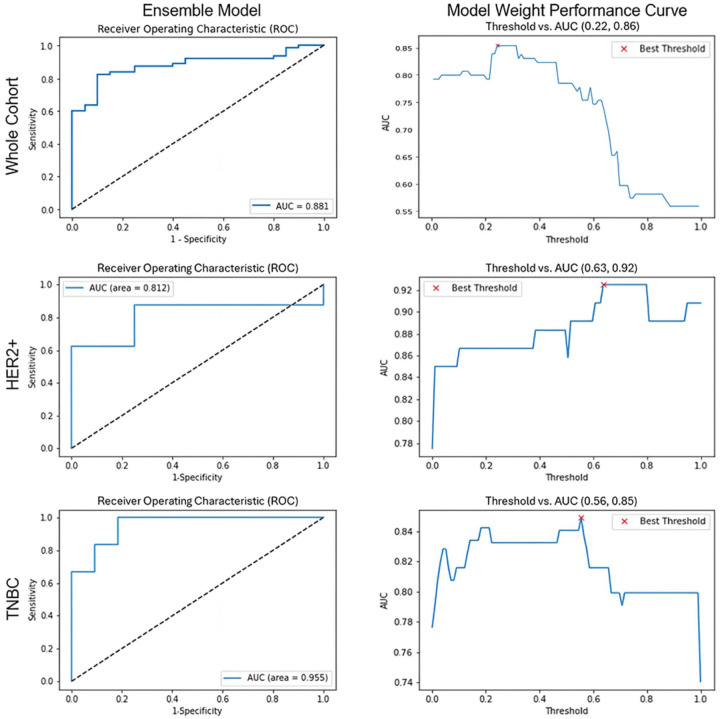
The ROC AUCs for the highest-performing ensemble models with optimized features for each subgroup. Graphs on the right indicate the optimal combination threshold for clinical feature and graph feature models. AUC ROC, area under the receiver operator characteristic curve.

In comparison, the TNBC group combined the SVM clinical model with the KNN_mean_ graph model at a threshold of 0.56 (Figure 5). Each model performed optimally using a different number and set of features. Table 3 indicates the highest-performing ensemble models and their feature sets. Among clinical models, inflammatory status was suggested in all high-performing models. Graph models frequently indicated “Density Neighbors in Distance 39 Mean, Disorder, Std Dev” as an essential feature in high-performing models.

**Table 3.** Optimal features for the highest performing models.

Ensemble classifier	AUC	Input features
XGBoost + GNB_min_	0.881	Age (⩽50 or >50), CCF_10_, clinical tumor staging, DBS_10_, DBS_70_, DND-MMR_0_, DND-DIS_0_, DND-STD_39_, DDN-MMR_13_, DDN-DIS_13_, HER2 status, inflammatory status, laterality, MCP_10_, MCP_20_, MCS_10_, multifocality, nottingham grade, OF_10_, OF_20_, OF_100_, PR status, VMD-MMR
RFC + KNN_median_	0.812	Backbone neoadjuvant therapy, Nottingham grade, STD-CP_30_, VMD-MMR
SVM + KNN_mean_	0.955	Clinical tumor staging, DBS_20_, DBS_40_, DND-STD_39_, DDN-MMR_13_, inflammatory status, Nottingham grade

Input features for optimal ensemble models.

AUC, area under the receiver operating characteristic curve; CCF, clustered cell fraction; CP, cluster persistence; DBS, Davies–Bouldin score; DDN, density distance for neighbors; DIS, disorder; DND, density neighbors in distance; GNB, Gaussian Naïve Bayes; HER2, human epidermal growth factor receptor 2; KNN, K-nearest neighbor; MCP, median cluster persistence, MCS, median cluster size; MMR, min–max ratio; OF, outlier fraction; PR, progesterone receptor; RFC, random forest classifier; STD, standard deviation; SVM, support vector machine; TNBC, triple-negative breast cancer; VMD, Voronoi max distance; XGBoost, extreme gradient boosting.

Performance metrics for each ML classifier were also identified and compared. LR ML models demonstrated moderate performance with clinical and graph features. Clinical ML algorithms demonstrated relatively low scores from the LR model (AUC = 0.839). Graph ML algorithms varied with the LR_weighted-mean_ classifier demonstrating modest performance (AUC = 0.781). The graph KNN_mean_ model demonstrated a high AUC with high accuracy (AUC = 0.924, Acc = 0.881; Table 4).

**Table 4.** Highest performance scores for classifiers.

Group	Classifier	AUC	Acc	Pre	F1	Sn (%)	Sp (%)
Clinical	KNN	0.751	0.723	0.833	0.813	79.3	50.0
	LR	0.839	0.814	0.300	0.429	75.0	82.0
	RFC	0.545	0.583	0.714	0.666	62.5	50.0
	SVM	0.850	0.759	0.877	0.833	79.3	65.0
	XGBoost	0.856	0.843	0.917	0.894	87.3	75.0
Graph	GNB_weighted-per-core_	0.807	0.116	0.095	0.174	100	2.50
	KNN_mean_	0.924	0.882	0.750	0.857	100	81.8
	LR_weighted-mean_	0.781	0.583	0.636	0.736	87.5	0.00
	RFC_max_	0.719	0.667	0.833	0.714	62.5	75.0
	SVM_median_	0.878	0.705	0.600	0.545	50.0	81.8
	XGBoost_mean-min-max_	0.674	0.647	0.500	0.400	33.3	81.8

Highest performance metrics for each classifier within clinical and graph feature groups.

Acc, accuracy; AUC, area under the receiver operating characteristic curve; GNB, Gaussian Naïve Bayes; KNN, K-nearest neighbor; LR, logistic regression; Pre, precision; RFC, random forest classifier; Sn, sensitivity; Sp, specificity; SVM, support vector machine; XGBoost, extreme gradient boosting.

Subgroup statistical analysis was conducted to compare clinical, graph, and ensemble models. Within the whole cohort group, the ensemble model (AUC = 0.881) indicated a statistical difference from the graph model (AUC = 0.731, *p* = 0.023). Statistical significance was also demonstrated between the clinical model (AUC = 0.561) and the graph model (AUC = 0.924, *p* = 0.026) as well as between the clinical (AUC = 0.561) and ensemble models (AUC = 0.955, *p* = 0.006) within the TNBC subgroup. There was a trend toward statistical significance between clinical and graph models in the whole cohort (*p* = 0.077; Table 5). No other comparisons were statistically significant.

**Table 5.** Statistical comparison of top model performances.

Group	Model	AUC	95% CI	Comparison	ΔAUC	*p*-Value
Whole cohort	Clinical	0.856	(0.776–0.936)	Graph	0.125	0.077
	Graph	0.731	(0.628–0.827)	Ensemble	0.150	0.023*
	Ensemble	0.881	(0.807–0.950)	Clinical	0.025	0.124
HER2+	Clinical	0.719	(0.296–1.000)	Graph	0.062	0.777
	Graph	0.781	(0.455–1.000)	Ensemble	0.031	0.704
	Ensemble	0.812	(0.501–1.000)	Clinical	0.093	0.662
TNBC	Clinical	0.561	(0.288–0.846)	Graph	0.363	0.026*
	Graph	0.924	(0.800–1.000)	Ensemble	0.031	0.427
	Ensemble	0.955	(0.827–1.000)	Clinical	0.394	0.006*

Delong test was performed to compare model AUC values.

* Statistically significant difference (*p* < 0.05).

AUC, area under the receiver operating characteristic curve; CI, confidence interval; HER2, human epidermal growth factor receptor 2; TNBC, triple-negative breast cancer.

## Discussion

The results of this study indicate that graph features obtained from TILs are predictive markers of pathological response in BC treated with NST. TILs have been studied across several malignancies.^40–42^ In BC, TILs are prognostic markers and have also been shown to predict response to systemic therapies.^15,16,18,20,43–47^ Previous reports have explored different assessment methods to evaluate TILs as biomarkers, such as gene expression analysis, VTAs, and computational TILs assessment (CTA). West et al. investigated gene microarrays yielding an eight-gene signature in estrogen-receptor-negative patients treated with NST. The results showed that the following TIL genes predicted pCR after NST: CD19, CD3D, CD48, GZMB, LCK, MS4A1, PRF1, and SELL. Unsupervised clustering analysis output three centroids associated with gene expression levels: (1) highly enriched TILs, (2) intermediate TILs, and (3) low TILs. Specimens with highly enriched TILs were significantly associated with a pCR and observed in 74.2% of cases. In multivariate models, a binary classification combining low and intermediate TILs into one group versus high TILs showed a pCR prediction accuracy of 70.3%.^43^

CTA has gained widespread interest, particularly with increasing computational capabilities and advancements in AI.^48^ Saltz et al. developed a DL method using two CNNs to map TILs. Data were collected from The Cancer Genome Atlas (TCGA), containing 13 tumor types.^49^ First, the CNNs yielded high accuracy, identifying lymphocyte-infiltrating regions in lung adenocarcinoma patches (AUC = 0.954), thus demonstrating the promise of CTAs for automated analysis. Second, affinity propagation was employed to characterize the TIL spatial organization (i.e., TIL clusters). The results showed distinct clustering patterns in various neoplasms, suggesting TILs as a new parameter for tumor characterization. Lastly, when age and gender were included as clinical covariates in multivariate analysis, there was a significant association between TIL cluster indices and OS across several tumor types. This suggests that CTA spatial analysis of TILs can be extended to prognostic modeling. The same research group conducted CTA and survival analysis in BC using data from the TCGA and the Carolina Breast Cancer Study.^26^ A computer vision pipeline comprised a ResNet34 architecture to detect tumor regions, followed by lymphocyte classification with a VGG16 model. The spatial organization of the TILs was mapped, and spatial patterns were scored using a rubric describing high-level tumor-immune interactions. Spatial features included intratumoral strength, TIL deserts, TIL forests, peritumoral strength, and tertiary lymphoid aggregates. The results showed that increased peritumoral and iTIL features (i.e., “forests”) were associated with prolonged survival. Meanwhile, the absence of iTILs (i.e., “deserts”) was linked to higher BC recurrence rates.^26^ Overall, this study demonstrates the growing utility of CTA to gain clinical insight and potentially help direct surveillance practices in BC. An investigation by Makhlouf et al. examined 2231 patients with luminal early-stage BC treated with hormonal therapy; spatial-level features from sTILs and iTILs were extracted to determine association with patients’ outcomes. Increased TIL count and proximity to stromal and tumor cells were associated with poor clinical outcomes.^25^ These prior studies demonstrate the growing trend in using AI to characterize the role of spatial TILs in predicting treatment response, characterize the tumor microenvironment, and quantify the risk of BC recurrence and/or death.

Compared to previous studies, our research proposed a new method to quantify the spatial distribution of TILs using a computer vision pipeline. We employed a spatial analysis method using quantitative imaging to extract TIL parameters. Utilizing a CNN-based segmentation and classification framework, ROIs in the CNB were delineated, and coordinates of TILs were employed to extract TIL spatial biomarkers. We then employed ML models to identify the most relevant features for distinguishing pCR from non-pCR, demonstrating strong classification performance. Notably, statistically significant differences in performance were demonstrated between clinical and ensemble as well as clinical and graph models in the TNBC group. Although these findings emphasize that TIL spatial characterization may supplement clinical information, the predominance of HR-positive disease may have affected TIL-level spatial metrics, limiting model performance. HR-positive disease demonstrates lower levels of TIL infiltration and immunogenicity, potentially reducing the impact of certain spatial metrics such as clustering or neighboring patterns. Nonetheless, our results corroborate previous reports in terms of TILs biomarkers of NST response. Here, we focus on a quantitative imaging approach using computationally derived spatial parameters (i.e., graph features) to measure TILs in BC. Within this context, we hypothesize that graph features are associated with qualitative descriptors of TILs’ spatial organization described in the VTA literature. These include, for example, density (e.g., weak/low, moderate/intermediate, dense/high), distribution (e.g., count/mm^2^), “forests,” and “deserts.” Graph features quantify TIL-TIL distances and clustering patterns and, to a lesser extent, their activity. These parameters may be useful in understanding intratumoral heterogeneity and biology. In so doing, these can be used in future studies for validation on external datasets and implementation into future prospective cohort studies and/or clinical trials.

The growing collection of CTA studies has yielded a need to standardize AI methods, develop disease-specific applications, and aggregate data to inform clinical practices.^48^ Protocol development for CTA has been a priority for the IBWG.^48^ Amgad et al. presented key issues for CTA and proposed initial frameworks to guide its research and development. Challenges include (1) consensus of ground-truth labels (i.e., identification of TILs within a ROI), (2) underperformance of segmentation algorithms due to suboptimal threshold settings of the H&E stains (classification errors), and (3) variations in imaging protocols (e.g., WSI resolution, magnification, and artifacts).^48^ Important frameworks to address some of these challenges include guidelines by the IBWG to prepare and stain tissue samples and methods to assess TILs in the stromal and intratumoral compartments. Standardizing imaging formats, such as DICOMS, has also been put forward to mitigate interoperability issues.^48^ Shifting practices from VTA to computer-driven systems will necessitate robust data training in the future. This training will include validation of ground-truth inputs (e.g., multi-user inputs), appropriate sample power to build confidence in the model, the inclusion of diverse and global datasets, and WSIs acquired from various digital pathology imaging systems.^48^

### Study limitations and future perspectives

To enable clinical translation of these findings, external validation across independent, multicenter cohorts is essential. Collaborations with other hospitals would allow inclusion of diverse patient demographics, as well as variations in imaging scanners, acquisition protocols, and clinical procedures, thereby enhancing model robustness and generalizability. A large subset of patients were HR-positive, where their lower immune activity could potentially affect predictive performance in the whole cohort analysis. Increasing representation of underrepresented molecular subtypes, including TNBC and HER2+, is essential to increase the reliable performance of models. Future studies could implement TIL-based models with additional features such as radiomic (computed tomography, magnetic resonance) or genetic data (gene assays) and incorporate immune cell subpopulations (i.e., CD4+, CD8+) to refine the prediction of therapy response.^50^ Beyond systemic therapy, TIL-based spatial analysis could be extended into radiotherapy or combined strategies, expanding their clinical utility.^51^ These efforts would support multicenter prospective studies and potentially, eventual clinical trials.

## Conclusion

In conclusion, this study’s findings suggest that ML-driven predictive models using clinical and TIL-based spatial features distinguish pCR from non-pCR in patients receiving NST. This offers insight into using the spatial distribution of TILs as a potential biomarker of NST response that may help tailor treatment regimens in future clinical practice.

## Supplemental Material

sj-docx-1-tam-10.1177_17588359261417762 – Supplemental material for A computer vision method to evaluate tumor-infiltrating lymphocytes and multiparametric modeling of neoadjuvant systemic therapy response in breast cancer Supplemental material, sj-docx-1-tam-10.1177_17588359261417762 for A computer vision method to evaluate tumor-infiltrating lymphocytes and multiparametric modeling of neoadjuvant systemic therapy response in breast cancer by Mateusz Bielecki, Fang-I Lu, Angeline Vo, Eileen Rakovitch, Katarzyna J. Jerzak, Roberto Salgado, Raffi Karshafian and William T. Tran in Therapeutic Advances in Medical Oncology

## Declarations

**Ethics approval and consent to participate:** All procedures involving the collection of tissue were conducted in accordance with the ethical standards of the institutional review board and have been approved by Sunnybrook Health Sciences Centre Research Ethics Board (Project Number: 2318). This was a retrospective study, and data were anonymized; and patient consent was waived.

**Consent for publication:** Not applicable in this case.

**Availability of data and materials:** The data curated and analyzed in this study are not available to the public; however, they may be made available upon reasonable request.
